# Diversity and Distribution of Deep-Sea Cetaceans in the Northern South China Sea Based on Visual and Acoustic Surveys

**DOI:** 10.3390/ani15192802

**Published:** 2025-09-25

**Authors:** Liang Fang, Xinxing Wang, Yujian Chen, Yuezhong Wang, Xinrui Long, Wentao Lu, Hancheng Zhao, Zhao Zhen, Kunhuan Li, Qilin Gutang, Tao Chen

**Affiliations:** 1 Sanya Tropical Fisheries Research Institute, Sanya 572000, China; wangxinxing@scsfri.ac.cn; 2 South China Sea Fisheries Research Institute, Chinese Academy of Fishery Science/Guangdong Provincial Key Laboratory of Fishery Ecology and Environment, Guangzhou 510300, China; chenyj20180910@163.com (Y.C.); wangyuezhong@scsfri.ac.cn (Y.W.); 3 Marine Biology Institute, Shantou University, Shantou 515063, China; 23xrlong@stu.edu.cn (X.L.); 23wtlu@stu.edu.cn (W.L.); 20hczhao@stu.edu.cn (H.Z.); 19zzheng@stu.edu.cn (Z.Z.); 18khli@stu.edu.cn (K.L.); kriskylin@stu.edu.cn (Q.G.)

**Keywords:** cetaceans, passive acoustic monitoring, South China Sea, deep-sea ecosystem

## Abstract

Cetaceans are essential for maintaining the balance and stability of deep-sea ecosystems. However, environmental challenges and limited funding have resulted in a marked lack of data on species diversity and the conservation status of deep-sea cetaceans. In this study, we undertook two research expeditions in the deep-water regions of the northern South China Sea, employing an integrated visual and acoustic survey approach. In total, 28 cetacean encounters, involving 12 species and more than 1000 individuals, were documented through visual observation, while acoustic monitoring recorded 53 detections. These findings demonstrate that the deep-sea waters of the northern South China Sea have relatively high cetacean biodiversity and constitute significant habitats for these marine mammals. Nevertheless, this area also experiences intensive human activities, with fisheries, maritime traffic, and oil and gas extraction posing primary threats to local cetacean populations. Improving the management of human activities in this marine zone is essential for ensuring the effective protection of cetacean species and their critical habitats.

## Simple Summary

Cetaceans serve a critical role in deep-sea ecosystems. However, the challenging environmental conditions and limited funding have led to a significant lack of data on the species diversity of deep-sea cetaceans. This study combined visual and acoustic survey methods to investigate whales and dolphins in the deep-water regions of the northern South China Sea. The findings reveal remarkably high cetacean biodiversity in these areas. Nonetheless, cetaceans in the South China Sea are facing severe anthropogenic pressures, including fishing activities, shipping traffic, and oil and gas exploration. Establishing and enforcing robust conservation policies is imperative to ensure the long-term survival of whales and dolphins in this region.

## 1. Introduction

Marine mammals are recognized as flagship species in marine ecosystems and are critical for maintaining the biodiversity and stability of marine environments [1,2,3]. However, the unique behavioral patterns of cetacean species and the high costs of marine surveys mean that most deep-dwelling marine mammals remain insufficiently studied [4]. Given the rapid pace of ocean exploitation and development, population surveys and distribution mapping of marine mammals are essential for species conservation and the protection of biodiversity in their respective habitats [5,6,7,8]. While coastal cetaceans have received relatively substantial research attention, offshore and deep-sea cetacean species have long been understudied due to environmental challenges and funding limitations, as well as their elusive behavior [9].

Deep-sea cetaceans play a vital role in ocean ecosystems [10]. When a whale dies, its massive carcass sinks to the seafloor and becomes a vital nutrient source for these ecosystems, a process known as “whale fall”. Decomposition can persist for decades, providing food and habitats for deep-sea organisms [11,12,13]. Furthermore, deep-sea cetaceans enhance the “biological pump” effect through vertical migration activities, such as diving to hunt and returning to the surface to breathe, which contributes to carbon transfer from surface waters to the deep ocean [14,15]. Additionally, cetacean excrement, rich in iron and nitrogen, stimulates phytoplankton growth, indirectly boosting the ocean’s carbon sequestration capacity [16,17,18,19]. Through their migratory and predatory behaviors, cetaceans influence prey distribution via their effect on fish and cephalopod populations, thereby maintaining the stability of marine food webs [19,20,21].

The South China Sea supports relatively high cetacean diversity, with 30 cetacean species documented in the region based on stranding data [22,23]. However, vast areas of its waters remain data-deficient zones in cetacean research. The lack of population data on dolphins and whales in these waters severely hampers effective conservation and management efforts. Recent research expeditions focusing on cetaceans in key areas of the northern and middle South China Sea have indicated that species richness is exceptionally high in the surveyed regions [24,25,26,27,28,29]. Nevertheless, the South China Sea is subject to intensive human activities, serving as a key international shipping route, major fishing ground, and significant zone for offshore oil and gas exploration [30]. Rising anthropogenic pressures, such as maritime traffic, overfishing, and resource extraction, are increasingly threatening the survival of these species [31]. Addressing these threats requires urgent, species-specific research and robust data collection to guide evidence-based conservation policies and mitigate the risks to marine biodiversity.

Which cetaceans are distributed in the deep-sea waters of the northern South China Sea, and what threats do they face? This is an important issue that requires focused attention against the backdrop of the South China Sea’s rapid development and utilization. Visual and acoustic survey methods are the most widely employed approaches in cetacean research for assessing population sizes and habitat distributions [32,33,34,35,36]. Conducting cetacean surveys in the South China Sea is essential for understanding and advancing the conservation of marine biodiversity in the region. The objectives of this study were to investigate and document the diversity and distribution patterns of deep-sea cetaceans in the northern South China Sea, evaluate their conservation status under existing threats, and provide foundational data to support the development of management and protection strategies by the relevant authorities.

## 2. Materials and Methods

### 2.1. Survey Area

**Figure 1:**
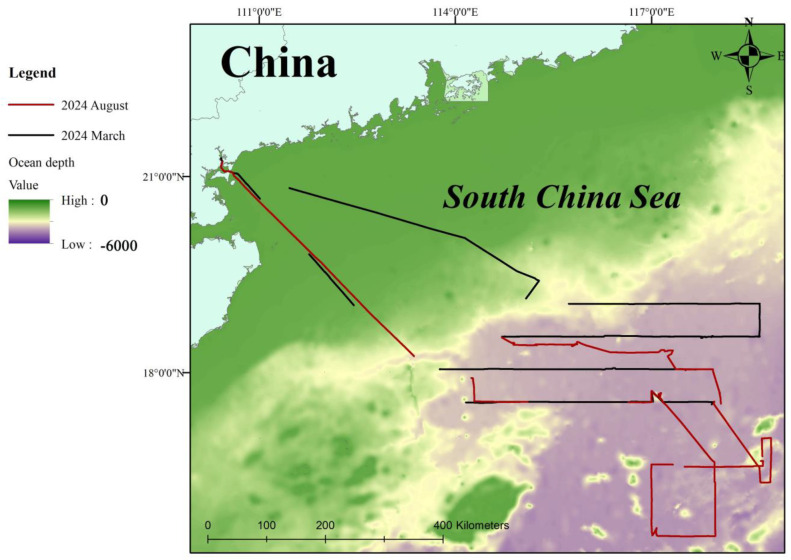
Map of the survey area in the northern South China Sea and the survey track.

The research area was located in the deep waters of the northern South China Sea (Figure 1). With depths reaching nearly 4000 m and averaging over 3500 m, it is one of the deepest areas in this body of water. This area lies in a major maritime traffic artery with the Taiwan Strait to the north and the Bashi Channel, both of which are important trade channels connecting the South China Sea with the Pacific Ocean, and shipping activities are very frequent. The northern part of the South China Sea is an important area for fishing and oil and gas production, and thus experiences intense human activities.

### 2.2. Visual Survey

For the visual survey, the standard line transect survey method was adopted and was designed according to the principles of distance sampling. A 300-ton iron-hulled vessel served as the research platform, with the ship’s top, standing at approximately 8 m above water level, serving as an observation deck. At any one time, the observation team consisted of three members, namely, two primary observers and one data recorder. The two primary observers were responsible for scanning both port and starboard sectors forward of the beam using 7 × 50 Fujinon binoculars (FUJIFILM, Tokyo, Japan), while the recorder documented sighting events and conducted supplementary naked-eye observations. The personnel were rotated every 20 min to prevent fatigue, with the full observation team comprising eight members, all possessing extensive cetacean observation experience. The vessel maintained a cruising speed of 6–8 knots during operations. Daily observations commenced at 07:30 h and concluded at 19:00 h. When cetaceans were sighted, the recorder documented the species, time, group size, global positioning system (GPS) coordinates, animal-vessel distance, sea state, angle, and water depth. Simultaneously, the vessel decelerated to approach the cetaceans for photographic documentation.

### 2.3. Passive Acoustic Survey

For the acoustic survey, an autonomous miniature stereo acoustic event data logger (A-tag, ML200-AS2; Marine Micro Technology, Saitama, Japan) was employed. This acoustic recorder has been extensively used for detecting the Yangtze finless porpoise (*Neophocaena asiaeorientalis asiaeorientalis*) during both mobile and stationary surveys [37,38,39]. Detailed specifications of the A-tag are provided in Akamatsu et al. [38]. The towed-type A-tag comprised two external ultrasonic hydrophones, a preamplifier (+60 dB), a band-pass filter (55–235 kHz), 128 MB of flash memory, and batteries. Each hydrophone had peak sensitivity at approximately 120 kHz (210 dB re 1 V/μPa), with a frequency response of 100–160 kHz within 5 dB. The hydrophones were spaced 17 cm apart. The data logger had a dynamic range of 129–157 dB (peak-to-peak, re 1 μPa). The detection range was highly dependent on the clicks emitted by animals and the level of background noise. As a reference, Akamatsu et al. [38] reported a detection range of approximately 300 m for the Yangtze finless porpoise in the Yangtze River. During the survey, the A-tag was towed approximately 100 m behind the survey boat using a nylon rope. Two iron bars (approximately 500 g each) were attached to the front of the A-tag to submerge the device and minimize surface noise interference. The A-tag working start time was manually set and synchronized with a GPS (eTrex 30×; Garmin, Schaffhausen, Switzerland).

### 2.4. Acoustic Data Analysis

**Figure 2:**
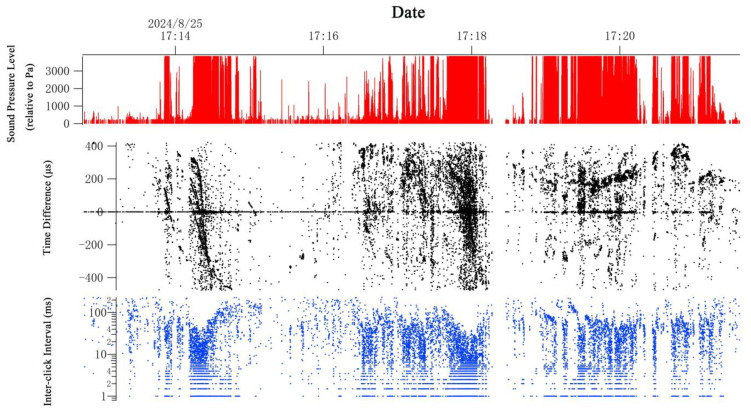
Example of echolocation clicks from a group of odontocetes. The vertical axes, respectively, show the echolocation click receive level (sound pressure level [SPL]), time difference (TD), and inter-click intervals (ICI).

A-tag data were processed using a customized program developed in Igor Pro 5.01 software (WaveMetrics, Portland, OR, USA). The extracted pulse event data comprised the sound pressure level (SPL) of the pulse, the time difference (TD) in pulse arrival between the two hydrophones, and the inter-click interval (ICI). These parameters can be used to identify vocalizations and track individuals. The SPL, TD, and ICI data are illustrated in Figure 2. Cetacean echolocation typically involves sequences of high-intensity clicks, known as click trains, ranging from several to hundreds of pulses, with ICIs spanning milliseconds to tens of milliseconds. Given that the survey boat was moving faster than the swimming cetaceans, the TD values corresponding to pulses from a vocalizing animal passing the A-tag exhibited a characteristic pattern. As the animal passed by the A-tag, the bearing angle changed, causing the TD to shift from positive to negative values (Figure 2). This shift generated a distinct trace in the TD plot, representing the passage of an individual animal. The detection time of the animal was defined as the point at which the TD was nearly equal to zero, and this time point was used to identify the position of the detected animal by matching it with the corresponding GPS track of the survey routine. While the vessel was moving, acoustic detections occurring within a 10 min interval were considered part of the same detection event. If the survey boat was following the animals, the entire tracking time constituted a single detection event.

### 2.5. Data Analysis

The position of visual and acoustic detections was presented by ArcMap version 10.3. Statistical analyses were conducted in SPSS (version 16.0; SPSS Inc., Chicago, IL, USA) with the significance level set at *p* < 0.01.

## 3. Results

### 3.1. Visual Sightings of Cetaceans

**Figure 3:**
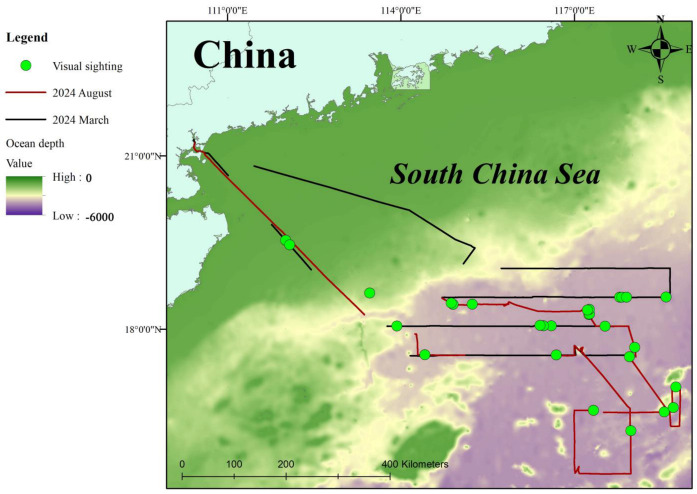
Location of the cetacean visual sighting events in the northern South China Sea.

This study encompassed two surveys. The first was conducted from 25 March to 7 April 2024, lasted 13 days, and covered 2156 km; the second voyage was undertaken between 15 August and 27 August 2024, lasting 12 days and spanning 1859 km. During the visual expedition, 28 cetacean sighting events were recorded, with 27 involving toothed whales and 1 a baleen whale (Table 1) covering 2739 km. The encounter rate of cetaceans is 1.02 per 100 km. The positions of these visual sightings are shown in Figure 3. The largest toothed whale species encountered was the sperm whale (*Physeter macrocephalus*). The most frequently encountered and numerically abundant species was the pantropical spotted dolphin (*Stenella attenuata*). Pantropical spotted dolphins, Risso’s dolphins (*Grampus griseus*), pilot whales (*Globicephala* spp.), and Indo-Pacific bottlenose dolphins (*Tursiops aduncus*) were consistently observed in large groups, typically exceeding 50 individuals. Additionally, two sightings of groups of the relatively rare beaked whales (*Ziphiidae*) were documented, each group consisting of four individuals. In 10 sightings, the cetacean species could not be identified due to the small group size and the short surfacing durations.

**Figure 4:**
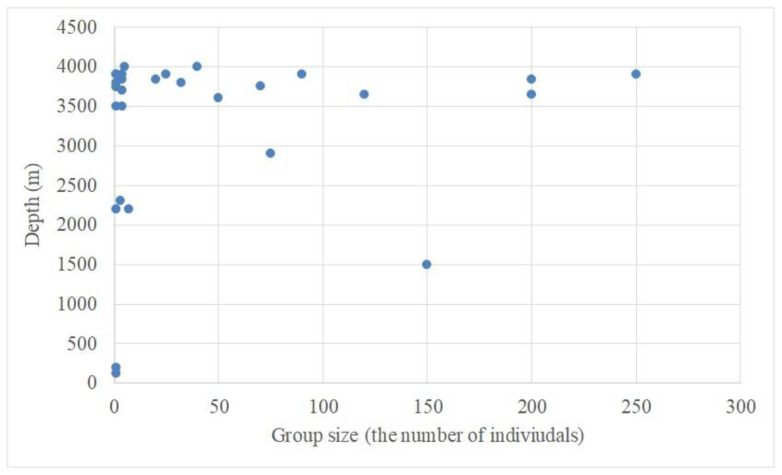
The sizes of the sighted cetacean groups and the depth of the sighting locations.

Of the 28 cetacean sightings, only 2 were recorded at depths < 500 m (Figure 4). The shallowest occurrence involved Bryde’s whale (*Balaenoptera brydei*) at a depth of approximately 120 m. Most sightings (75%) were at depths between 3500 and 4000 m.

### 3.2. Acoustic Survey Detection

**Figure 5:**
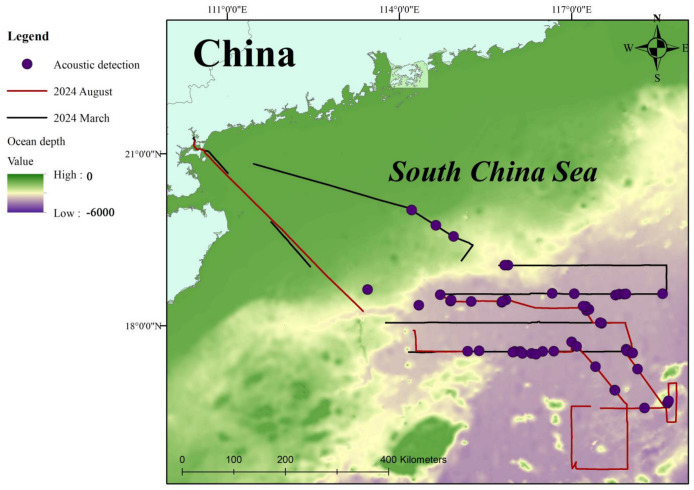
Location of cetacean acoustic detection events in the northern South China Sea.

In the acoustic survey, a total of 52 acoustic events were detected, of which 14 were confirmed by both visual and acoustic methods. During the first survey, 23 cetacean vocalizations were detected. Eighteen acoustic signals occurred in daylight (06:00–19:00 h), while five were recorded at night (19:00–06:00 h), with the vessel under navigation throughout the night. In the second survey, 30 cetacean acoustic signals were identified, comprising 21 daytime detections (06:00–19:00 h) and 9 nocturnal ones (19:00–06:00 h). In this survey, the vessel engine was shut down at night. The data concerning the acoustic detection of cetaceans is presented in Table 2, and the locations of the detections are depicted in Figure 5.

## 4. Discussion

### 4.1. Cetacean Diversity in the Deep-Sea Area of the Northern South China Sea

Although several studies have recently been conducted on cetaceans in the South China Sea, relevant information remains scarce [18,24,25,26,27,28,29]. The growing anthropogenic pressures in this region make it an urgent task to assess the diversity and conservation status of local whales and dolphins for developing effective conservation strategies [31]. In this study, we investigated cetacean diversity in waters nearly 4000 m deep in the northern South China Sea through integrated visual and acoustic surveys. The findings revealed remarkably rich cetacean biodiversity in this deep-sea area, with the pantropical spotted dolphin, Risso’s dolphin, pilot whales, and beaked whales being the most frequently encountered species. The pantropical spotted dolphin was the most frequently encountered and numerically dominant cetacean species, with large groups sighted during both survey voyages. This species exhibits one of the highest bycatch rates among cetaceans in the South China Sea—likely linked to its feeding ecology, particularly in fisheries using vessels with light attraction and drift nets [40,41,42]. Risso’s dolphin (*Grampus griseus*), a cosmopolitan species, was also regularly observed during the surveys. This species exhibits a broad global distribution, primarily inhabiting tropical and temperate waters [43]. Within the Indo-Pacific region, it is frequently reported in the waters east of Taiwan, predominantly occurring at depths between 300 and 1500 m [44]. Previous surveys in the South China Sea have also documented sightings of this species [28,29]. The short-finned pilot whale (*Globicephala macrorhynchus*), a large cetacean commonly sighted in the South China Sea, was repeatedly documented by Liu et al. [27]. Current research indicates that this population does not undergo long-distance migration, suggesting that the South China Sea may constitute a critical regional habitat [27]. Accordingly, this resident species should be prioritized in regional conservation strategies. Previous studies suggest that during the spring internal tide period, large-amplitude internal waves develop in the Luzon Strait between Taiwan and the Philippine archipelago in the South China Sea. These waves sink near the Dongsha Plateau, uplifting nutrients and plankton to the upper ocean layers, potentially attracting pilot whales to forage in this region [45]. Sperm whales (*Physeter macrocephalus*), the largest species encountered in this survey, remain understudied in Asian waters despite frequent stranding records along China’s coast, including the South China Sea [26,46]. Liu et al. [23] proposed that the South China Sea represents a potential calving ground for sperm whales, based on a synthesis of multiple survey datasets. This possibility was reinforced by our direct observation of a cow-calf pair within a sperm whale group in the present survey. Furthermore, fisheries resource assessments show that purpleback flying squid (*Sthenoteuthis oualaniensis*) are abundantly present in these waters [47], and could thus provide a reliable prey base to support the local sperm whale population. Bryde’s whales (*Balaenoptera edeni*) were the only baleen whales sighted. Although strandings of these animals are frequently reported in the South China Sea and adjacent waters, their movement patterns remain poorly understood. While a seasonal aggregation is known to occur in the Beibu Gulf of Guangxi from October to approximately May, their whereabouts during other periods are unknown [48,49,50]. Recent sightings in Hong Kong and Shenzhen waters suggest that eastern Hainan and Guangdong coasts are important distribution areas for this species [51]. Studies have identified high cetacean diversity near the Zhongsha Islands, further confirming the rich abundance and diversity of whales and dolphins throughout the South China Sea [28,29].

### 4.2. Comparison Between the Visual and Acoustic Surveys

The acoustic survey in this study demonstrated superior detection efficacy compared to the visual survey, with a total of 53 detection events recorded. Acoustic techniques proved more effective in detecting cetacean presence overall. However, under favorable sea conditions, visual surveys still offer advantages in detecting animals at greater distances and in identifying specific species. The passive acoustic system employed in this study had an operational bandwidth of 55–235 kHz. This frequency range may exclude lower-frequency acoustic signals, such as sperm whale echolocation clicks, which exhibit peak frequencies below 20 kHz [52,53,54]; false killer whale (*Pseudorca crassidens*) and pilot whale clicks, where peak energy typically occurs below 50 kHz [55,56,57,58]; and most beaked whale clicks, which predominantly feature peak frequencies under 60 kHz [59,60,61].

Visual and acoustic surveys are the most commonly used methods for investigating offshore cetaceans and have long been widely applied in offshore cetacean survey practices. Within the 7.8 million km^2^ study area of the eastern temperate North Pacific, sperm whale abundance was acoustically estimated, with 45 distinct groups being localized. Acoustic technology proved capable of detecting the slow “clicks” produced by sperm whales from greater distances (up to 37 km). By extending the detection range and enabling nocturnal monitoring, acoustic technology significantly increased the number of sperm whales detected during line-transect surveys; nevertheless, visual observations remain essential for estimating group size [36]. Acoustic and visual surveys conducted in Gwaii Haanas demonstrated strong complementarity in detecting the presence and activity patterns of cetaceans, jointly identifying multiple species, while each method uniquely recorded others. However, both methods faced limitations with unidentified observations and vocalizations [62]. Considering the extended periods deep-diving animals spend submerged, we propose that acoustic surveys be used as an alert tool to guide visual surveys. This approach would significantly boost the animal detection rate during visual survey operations, which is of critical importance for identifying species, determining group size, and assessing population abundance.

### 4.3. Threats

As deep offshore waters lie beyond the public eye, the conditions and threats faced by cetaceans (whales and dolphins) in these remote marine areas often tend to be overlooked. The major threats to cetaceans in the deep offshore waters of the South China Sea include fishing activities, shipping, and oil and gas extraction.

#### 4.3.1. Fishing Activities

The South China Sea accounts for approximately 12% of global marine catches, ranking among the world’s top five fishing grounds. It hosts approximately 55% of global marine fishing vessels and serves as a primary fishing zone for neighboring countries/regions [63]. Marine fisheries hold historical significance in this region, with most bordering nations being major fishing producers and home to one-third of the world’s fishing population. Since the 1970s, marine fisheries have remained vital to these economies [64,65]. However, advancements in fishing gear, practices, and high-powered vessels have led to severe overcapacity, particularly in coastal waters. Fishing intensity now far exceeds sustainable yields, resulting in widespread overfishing [66,67]. Destructive methods, including bottom trawls, fine-meshed nets, and blast/electro-fishing, have degraded marine habitats and decimated fish stocks, driving resource depletion [68]. Over-fishing not only destroys marine ecosystems but also leads to a sharp decline in cetacean food sources. Additionally, these fishing operations result in a large number of cetaceans being caught as bycatch [24,41,42].

#### 4.3.2. Shipping

The South China Sea is one of the world’s busiest maritime regions. However, assessing the impact of its intensive shipping activities on cetaceans (whales and dolphins) remains challenging due to a lack of baseline ecological data. The primary impacts of shipping on pelagic cetaceans are underwater noise pollution and vessel collisions. These marine mammals rely heavily on acoustic signals for navigation, foraging, and social interactions, and ship noise masks their echolocation and communication frequencies, leading to failed prey detection and interrupted group coordination [69,70,71,72,73,74]. The disruption of cetacean sonar and communication systems by vessel noise has been documented globally; for example, in the North Sea, shipping noise was reported to cause hearing threshold shifts in cetaceans, forcing some species to alter migration routes to avoid acoustically degraded zones [75]. Arctic narwhals exhibit extreme noise sensitivity, performing rapid deep dives and ceasing social behaviors when vessels approach within 5 km. Importantly, these stress responses can accelerate energy depletion [76]. Vessel collisions are a leading cause of mortality for pelagic cetaceans. Approximately 45% of humpback whale deaths along the Atlantic coast are directly linked to ship strikes or propeller injuries [77], while annual collision mortality reached 4% for beluga whales in Canada’s St. Lawrence River [78].

#### 4.3.3. Oil and Gas Extraction

The impacts of current offshore oil and gas exploration and extraction activities in the South China Sea on cetaceans have long been overlooked due to a severe lack of assessment of these effects. The South China Sea is a regionally significant sea with relatively rich hydrocarbon resources; however, most of these resources are located in waters exceeding 500 m in depth [79,80]. As oil and gas operations advance into deeper waters, cetaceans inhabiting these marine zones are inevitably exposed to impacts from exploration and production activities [81,82]. In oil and gas exploration, seismic survey technology relies on airgun arrays, which emit high-intensity sound waves exceeding 200 dB re µPa every 10–12 s, lasting for weeks to months. This noise, comparable to underwater explosions, can propagate thousands of kilometers, severely disrupting cetaceans’ sound-dependent navigation, foraging, and reproductive activities [83,84,85,86,87]. In the Gulf of Mexico, it was shown that airgun noise reduced the foraging efficiency of sperm whales by 20% [88] and, regarding the responses elicited, both the received level and proximity to the source were reported to be important [89]. Meanwhile, oil and gas leaks can result in direct mortality or long-term physiological effects on marine cetaceans; for example, oil coating the bodies of dolphins led to thermoregulation failure and vision impairment, in addition to the toxin entry into the food chain [82,90].

### 4.4. Conservation and Management

Measures for enhancing the conservation and management of cetacean populations should include the following:

1. Prioritize comprehensive cetacean surveys in the South China Sea to document population size, distribution patterns, conservation status, and anthropogenic threats, particularly for large-bodied species or mass aggregations.
2. Strengthen conservation management frameworks by addressing impacts from fisheries, maritime traffic, and oil resource extraction. Current impact assessments remain inadequate. Consequently, enacting dedicated legislation for cetacean protection will empower law enforcement against violations.
3. Promote sustainable fisheries practices critical for long-term cetacean survival. This reduces bycatch and entanglement (e.g., from drift nets and purse seines, which are primary causes of non-natural mortality), while safeguarding prey resources and restoring fish stocks.
4. Intensify conservation outreach to deepen understanding among government agencies and public stakeholders regarding urgent threats and the imperative for immediate protection measures.

## 5. Conclusions

This study investigated cetaceans in the deep-sea region of the northern South China Sea through two surveys employing visual and passive acoustic methods. During the expedition, 28 cetacean sighting events were recorded, with 27 involving toothed whales and 1 a baleen whale. In the acoustic survey, a total of 52 events were detected, of which 14 were confirmed by both visual and acoustic methods. The results indicate that the deep-sea waters in the northern South China Sea host exceptionally rich cetacean biodiversity and serve as critical habitats for these marine mammals. Considering the growing human activities in the South China Sea, we propose implementing measures such as conducting additional scientific expeditions, enhancing habitat management, promoting sustainable fisheries development, and strengthening international cooperation to protect cetaceans in this region.

## Figures and Tables

**Table 1:** Summary of the information on cetacean visual sightings, including date, time, species, position, group size, depth, and sea state.

Date (Year-Month-Day)	Time (Hour-Minute)	Species	Longitude (°)	Latitude (°)	Group Size (Number of Individuals)	Depth (m)	Sea State
2024-03-26	09:14	Bryde’s whale (*Balaenoptera brydei*)	112.003	19.534	1	120	2
2024-03-26	10:23	Unidentified	112.071	19.454	1	200	2
2024-03-28	09:24	Pantropical spotted dolphin *Stenella attenuata*	116.683	17.551	40	4000	1
2024-03-29	15:20	Common dolphin (*Delphinus delphis*)	116.597	18.054	50	3600	1
2024-03-29	16:19	Risso’s dolphin (*Grampus griseus*)	116.462	18.054	70	3750	1
2024-03-29	17:11	Striped dolphin (*Stenella coeruleoalba*) Pantropical spotted dolphin Spinner dolphin (*Stenella longirostris*)	116.403	18.062	90	3900	1
2024-03-30	16:20	Unidentified	113.927	18.052	1	3800	1
2024-04-01	07:50	Pilot whale (*Globicephala* spp.)	117.779	18.550	200	3840	2
2024-04-01	09:20	Risso’s dolphin	117.818	18.548	20	3840	1
2024-04-01	10:24	Sperm whale	117.899	18.550	4	3840	1
2024-04-01	16:41	Striped dolphin	118.584	18.551	1	3740	1
2024-08-15	12:07	Unidentified	114.410	17.557	1	3500	4
2024-08-17	09:53	Unidentified	117.975	16.243	4	3500	2
2024-08-18	17:58	Unidentified	117.329	16.594	5	4000	2
2024-08-19	15:22	Unidentified	118.553	16.568	1	2200	2
2024-08-19	17:24	Unidentified	118.708	16.642	7	2200	2
2024-08-20	09:57	Unidentified	118.752	16.999	3	2300	3
2024-08-21	17:21	Spinner dolphin	117.950	17.522	75	2900	
2024-08-22	08:23	Beak whale	118.039	17.682	4	3900	1
2024-08-22	14:32	Pantropical spotted dolphin Spinner dolphin	117.528	18.046	250	3900	2
2024-08-22	18:40	Unidentified	117.248	18.249	1	3900	1
2024-08-22	19:00	Unidentified	117.260	18.258	1	3900	2
2024-08-23	07:27	Pantropical spotted dolphin	117.253	18.338	32	3800	2
2024-08-23	07:48	Striped dolphin	117.224	18.327	25	3900	2
2024-08-24	11:37	Beak whale	115.230	18.429	4	3700	2
2024-08-24	15:20	Pantropical spotted dolphin	114.899	18.424	120	3650	2
2024-08-24	16:35	Pantropical spotted dolphin Bottlenose dolphin (*Tursiops aduncus*)	114.867	18.449	200	3650	2
2014-08-25	17:02	Bottlenose dolphin	113.456	18.621	150	1500	2

**Table 2:** Summary of acoustic detection events for toothed cetaceans, including date, time, and position.

Event	Date (Year-Month-Day)	Time (Hour-Minute-Second)	Latitude (°)	Longitude (°)
1	2024-03-28	3:29:50	17.550	116.006
2	2024-03-28	4:21:30	17.550	116.104
3	2024-03-28	7:50:55	17.550	116.495
4	2024-03-28	9:26:30	17.553	116.687
5	2024-03-28	13:28:45	17.717	117.000
6	2024-03-28	14:34:45	17.636	117.085
7	2024-03-29	2:30:00	17.589	117.951
8	2024-03-29	16:26:00	18.054	116.462
9	2024-03-29	17:12:00	18.062	116.403
10	2024-03-31	1:32:00	18.353	114.334
11	2024-03-31	23:22:00	18.561	116.658
12	2024-04-01	2:15:00	18.551	117.045
13	2024-04-01	8:30:00	18.530	117.765
14	2024-04-01	9:30:00	18.544	117.826
15	2024-04-01	10:36:00	18.544	117.911
16	2024-04-01	11:51:00	18.550	117.944
17	2024-04-01	16:48:00	18.551	118.584
18	2024-04-01	22:57:00	19.051	115.888
19	2024-04-01	23:29:00	19.050	115.832
20	2024-04-03	12:32:00	19.551	114.939
21	2024-04-03	15:10:00	19.746	114.633
22	2024-04-03	18:52:00	20.014	114.209
23	2024-08-15	19:18:00	17.552	115.187
24	2024-08-15	21:38:15	17.559	115.387
25	2024-08-16	1:03:30	17.534	115.976
26	2024-08-16	2:04:00	17.516	116.140
27	2024-08-16	3:37:10	17.516	116.299
28	2024-08-16	3:50:00	17.502	116.376
29	2024-08-16	21:55:00	17.283	117.413
30	2024-08-17	1:31:50	16.874	117.750
31	2024-08-17	1:34:10	16.870	117.753
32	2024-08-19	13:06:00	16.564	118.268
33	2024-08-19	18:15:00	16.650	118.669
34	2024-08-19	21:52:00	16.689	118.686
35	2024-08-20	14:54:00	17.242	118.148
36	2024-08-21	17:40:00	17.557	117.96
37	2024-08-21	20:55:00	17.523	118.06
38	2024-08-21	23:30:00	17.523	118.06
39	2024-08-22	14:40:00	18.038	117.521
40	2024-08-22	15:29:00	18.048	117.498
41	2024-08-22	19:35:00	18.258	117.259
42	2024-08-23	3:30:00	18.278	117.3
43	2024-08-23	7:35:00	18.33	117.244
44	2024-08-23	8:06:30	18.333	117.2
45	2024-08-23	20:30:00	18.446	115.852
46	2024-08-24	0:00:00	18.405	115.781
47	2024-08-24	2:00:00	18.418	115.79
48	2024-08-24	12:35:00	18.418	115.247
49	2024-08-24	15:26:44	18.424	114.888
50	2024-08-24	16:39:00	18.443	114.899
51	2024-08-24	20:35:00	18.536	114.71
52	2024-08-25	17:12:00	18.625	113.442

## Data Availability

The data presented in this study are available on request from the corresponding author. Please contact the primary author for data requests.

## Abbreviations

GPS	Global positioning system
ICI	Inter-click interval
SPL	Sound pressure level
TD	Time difference
